# Bone Involvement in Rosai-Dorfman Disease (RDD): a Case Report and Systematic Literature Review

**DOI:** 10.1007/s11926-017-0656-6

**Published:** 2017-04-11

**Authors:** Birgit A. Mosheimer, Bastian Oppl, Shahin Zandieh, Michael Fillitz, Felix Keil, Klaus Klaushofer, Günter Weiss, Jochen Zwerina

**Affiliations:** 1 Department of Internal Medicine VI, Infectious Diseases, Immunology, Rheumatology, Pneumology, Medical University of Innsbruck, Anichstrasse 35, Innsbruck, Austria; 2 Ludwig Boltzmann Institute of Osteology at the Hanusch Hospital of WGKK and AUVA Trauma Centre Meidling, 1st Medical Department, Hanusch Hospital, Vienna, Austria; 3 Institute of Radiology and Nuclear Medicine, Hanusch Hospital, Vienna, Austria; 4 3rd Medical Department, Hanusch Hospital, Vienna, Austria

**Keywords:** Rosai-Dorfman disease, Bone involvement, Treatment strategies

## Abstract

**Purpose of Review:**

Rosai-Dorfman disease (RDD) is a rare histiocytic disorder typically presenting as painless cervical lymphadenopathy. Extranodal involvement is common and may also affect bones. Here, we present a patient with typical nodal disease and multifocal bone manifestations. Further, a systematic literature review was performed to better understand the phenotype, clinical course and treatment options of such patients.

**Recent Findings:**

RDD is a nonmalignant, classically sporadic histiocytosis. Nevertheless, increasing evidence also suggests familial forms of the disease. According to our literature review, bone involvement is exceedingly rare and heterogeneous. Clinical outcome in terms of mortality seems to be favorable in most cases. Currently, therapy strategies include surgical and immunosuppressive treatments, but the optimal treatment of osseous RDD remains to be defined.

**Summary:**

Patients with osseous RDD may present to rheumatologists with arthralgia or arthritis. Due to the rarity of the disease, diagnosis and treatment remain challenging.

**Electronic supplementary material:**

The online version of this article (doi:10.1007/s11926-017-0656-6) contains supplementary material, which is available to authorized users.

## Introduction

Rosai-Dorfman disease (RDD), also known as sinus histiocytosis with massive lymphadenopathy, is a rare histiocytic disorder typically presenting as painless cervical lymphadenopathy in adolescents and young adults [1, 2]. Constitutional symptoms and laboratory signs of inflammation may exist but are not mandatory.

RDD was long classified as a non-Langerhans cell histiocytic disorder (non-LCH). Non-LCHs were defined by the accumulation of histiocytes that do not meet the phenotypic criteria for the diagnosis of Langerhans cells (LCs) [3, 4]. Recently, a revised classification of histiocytic disorders and neoplasms of the macrophage-dendritic cell lineage has been proposed with RDD forming its own subtype (“R group”) due to its unique characteristics [5•]. In general, cell proliferation of the different histiocytic diseases can be either malignant or non-malignant; however, in RDD, histiocyte proliferation is presumably reactive and polyclonal [6].

Lymph node histology in RDD shows sinus dilatation due to typical histiocyte proliferation [7••]. Histiocytes in RDD exhibit emperipolesis, the non-destructive phagocytosis of lymphocytes and erythrocytes, which is the hallmark of the disease and required for diagnosis [7••, 8]. Immunohistochemical staining of RDD histiocytes is positive for CD68, CD14, HLA-DR, fascin, CD163 and S100 and typically negative for CD1a, distinguishing it from Langerhans histiocytosis [6]. In contrast, in Erdheim-Chester disease (ECD), another histiocytic disorder seen in adults, histiocyte staining is generally negative for S100 [9]. Infiltration of involved tissues with IgG4-positive plasma cells is common in RDD and differentiation from an IgG4-related disease may be an issue [10].

The clinical course of RDD is often benign, though lethal outcomes are also possible. Extranodal involvement is common and may occur in more than 40% of patients, sometimes without associated lymphadenopathy [1, 11]. The most frequent affected extranodal sites include the skin, nasal cavity and paranasal sinuses, eye and orbits and the central nervous system [2, 7••]. Synchronous involvement of extranodal sites is possible. Clinical outcome depends on the affected organs as well as on the number of extranodal sites involved [2].

Bone involvement occurs in less than 10% of cases [1]. There are also several reports about primary RDD of the bone without lymphadenopathy; the largest series describes 15 cases [12]. Clinical presentation includes pain or swelling but bone lesions may also be an incidental finding. On radiographs, skeletal lesions are typically lytic and intramedullary [13], sometimes with surrounding sclerosis. Purely sclerotic lesions—typically seen in ECD—are rare [14].

RDD patients may present to rheumatologists because of bone or joint pain. Due to the rarity of the disease, diagnosis and treatment are challenging. Here, we present a patient with typical nodal disease and multifocal bone manifestations. Further, a systematic literature review was performed to better understand the phenotype, clinical course and treatment options of such patients.

## Methods

### Case Report

A chart review of the presented RDD patient was performed and radiographic pictures as well as histology from bone and lymph nodes were retrieved. The patient gave written consent for the publication of this case report.

### Systemic Literature Review

An English literature search with the keywords “Rosai-Dorfman,” “Rosai and Dorfman” and “sinus histiocytosis with massive lymphadenopathy” was performed for the period between January 2000 and April 2015 using the PubMed database. A workup of the identified records according to the PRISMA (Preferred Reporting Items for Systematic Reviews and Meta-Analyses) scheme was conducted (Supplemental Fig. 1). Records where bone involvement could be ruled out by the abstract as well as full-text articles with insufficient description of bone involvement were excluded. Finally, 88 full-text articles covering 108 RDD patients with bone involvement were analyzed (Supplemental Text). All articles represent single case reports or small case series. Demographic and clinical characteristics and treatment and length of follow-up were retrieved from case reports where available.

The involved bones were grouped in the following regions: the cranium, facial bones, spine/sacrum, ribs, clavicle, sternum, scapula, humerus, radius/ulna, carpal bones, digits, pelvis, femur, tibia/fibula and tarsal bones. In 93 patients, RDD of the bone was histologically confirmed. The remaining cases reported imaging findings compatible with RDD of the bone while the histology was taken from the lymph nodes or other organs.

## Case Report

**Fig. 1:**
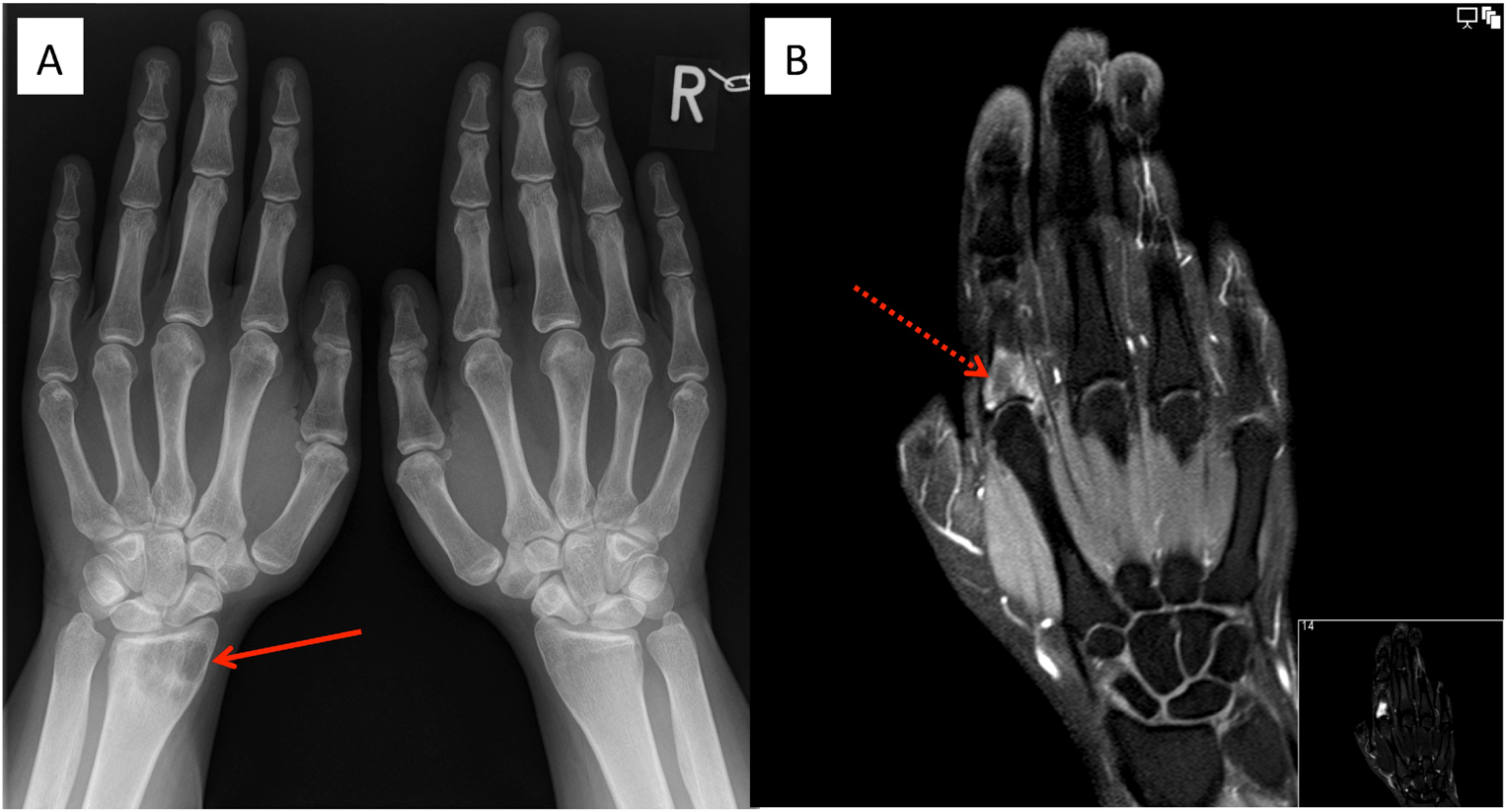
Bone involvement in Rosai-Dorfman disease (RDD). X-ray of the hands shows a lytic lesion (*arrow*) of the left distal radius (**a**). MRI demonstrated an additional mass lesion (*dotted arrow*) involving the proximal part of the right second phalanx with soft tissue extension (**b**)

A 29-year-old female patient presented with arthralgia in the hands and knees lasting for 1 year. Three months earlier, she was diagnosed with nodal Rosai-Dorfman disease after cervical lymph node excision when she presented with painless cervical lymphadenopathy. Physical examination revealed a dactylitis-like swelling of the second right digit and enlarged cervical lymph nodes. Laboratory testing showed significant signs of ongoing inflammation (C-reactive protein (CRP) 82 mg/l, ESR 106 mm/h, IgG 2502 mg/dl). Antinuclear antibodies, rheumatoid factor and anticitrullinated peptide antibodies were negative. Serologically, there was no evidence for an infection with CMV, HIV, hepatitis B or C, Borrelia burgdorferi or toxoplasmosa, whereas only EBV IgG was positive indicating previous infection with EBV. X-ray of both hands showed a lytic lesion of the left distal radius (Fig. 1a). MRI demonstrated an additional mass lesion involving the proximal part of the right second phalanx with soft tissue extension (Fig. 1b). Due to fracture risk, curettage of the radial lesion was performed. Histologically, typical signs of RDD were found. There was no mutation in BRAF V600E, which can be found in other histiocytic disorders [15].

Due to long-standing disease duration of more than 1 year, spontaneous remission was considered unlikely. Glucocorticoid therapy followed by addition of methotrexate (up to 25 mg weekly) was started. However, CRP levels remained elevated and follow-up MRI scans demonstrated slight progression of both the radial and metacarpal lesions. Therefore, rituximab (2 × 1000 mg) was administered in 2014 in addition to methotrexate. After 1 year of follow-up, the patient was free of symptoms and there was no disease progression on MRI scans. Due to the desire to become pregnant, immunosupressive medication was tapered and finally stopped without any change in clinical, serological or radiological findings. Nevertheless, inflammatory markers are still slightly elevated and nodal disease persists indicating chronic stable disease.

## Systematic Literature Review

One hundred and eight RDD patients with bone involvement were reported in 88 articles (Table 1). With 59 (55.1%) female and 48 (44.9%) male patients reported, gender distribution was balanced. Mean age of patients was 31.1 ± 19.8 years. Age ranged from three cases of newborns to a maximum of 79 years.

**Table 1:** Characterization of 108 RDD patients with bone involvement

	All (*n* = 108)	n/a, *n*
Age, mean ± SD	31.1 ± 19.8	1
Male, *n*	48 (44.9%)	1
Female, *n*	59 (55.1%)	
Primary RDD of the bone	67 (74.4%)	18
Lymphadenopathy	23 (25.6%)	
Number of bone regions involved, *n*	Number (%)	
1	76 (71.0%)	1
2	25 (23.4%)	
3	2 (1.9%)	
4	2 (1.9%)	
5	1 (0.9%)	
6	1 (0.9%)	
Top 3 osseus manifestations (*n* = 108)		
Cranium, *n* (%)	33 (30.6%)	
Facial bones, *n* (%)	24 (22.2%)	
Tibia, *n* (%)	19 (17.6%)	
Top 3 extraosseus manifestations (*n* = 80)		
Soft tissue, *n* (%)	27 (33.8%)	
Nodal disease, *n* (%)	23 (28.8%)	
Sinuses, *n* (%)	21 (26.3%)	

**Fig. 2:**
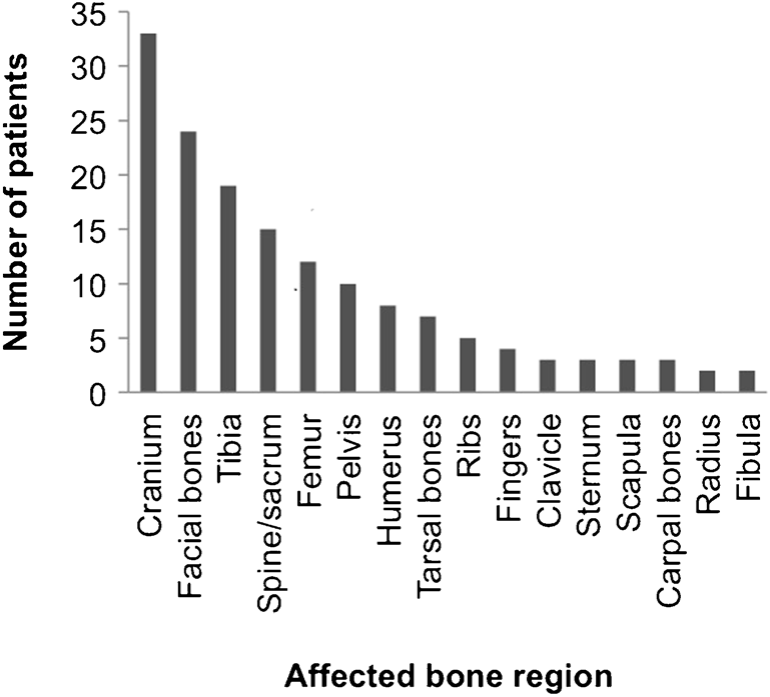
Distribution of bone manifestations in reported RDD patients (*n* = 108)

**Fig. 3:**
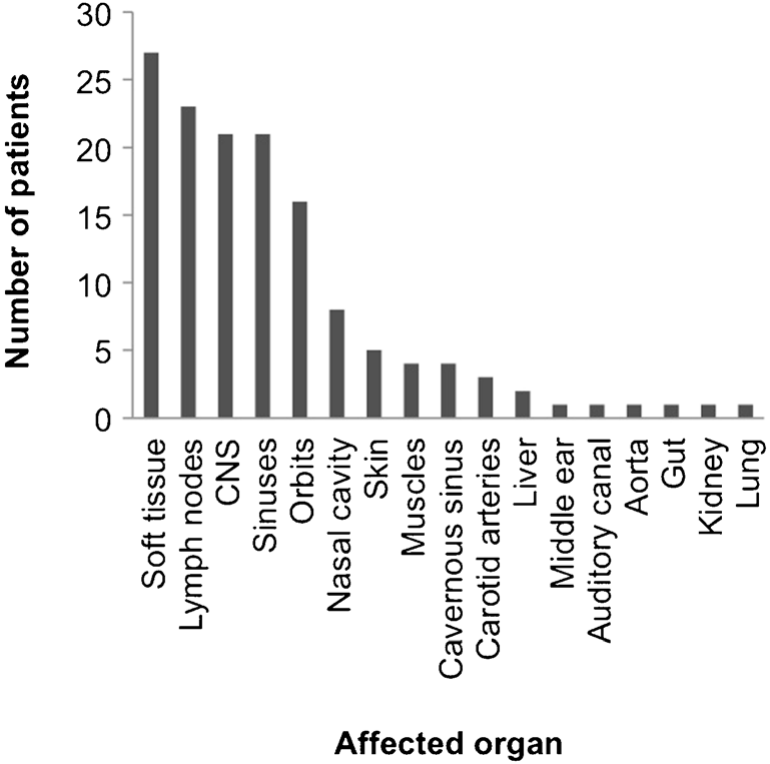
Distribution of extraosseus involvement (*n* = 80) in reported RDD patients

Sixty-seven individuals had primary RDD of the bone, defined as not developing lymphadenopathy during the disease course. Of those, 28 patients had lesions confined to the bone, whereas the rest showed involvement of one or more other organs (soft tissue 17, sinuses 13, central nervous system 11, orbit 9, nasal cavity 5, vessels 4, muscle 3, kidney 1). Lymphadenopathy was described in only 23 of cases of osseous RDD; while in 18 cases, information on lymph node involvement was not available. Other coexisting histiocytic disorders were not described in any case of osseus RDD at all. Ninety-seven percent of patients suffered from symptomatic disease. In the vast majority of patients, one (*n* = 76, 71.0%) or two (*n* = 25, 23.4%) bone regions were affected; while for a single patient, a total of eight affected bone regions, including the cranium, sternum, clavicle, ribs, fingers, pelvis, femur and tarsal bones were reported. The top three osseus manifestations were the cranium (*n* = 33), the facial bones (*n* = 24) and the tibia (*n* = 19) (Fig. 2). Imaging showed lytic lesions in 53 patients and mixed lytic and sclerotic lesions in 12 patients. Only one purely sclerotic lesion was reported. In 32 cases, this information could not be retrieved from the reports. RDD of the bone was histologically proven in 93 patients. Extraosseus manifestations were described in 80 (74.1%) patients, mainly affecting the soft tissue (*n* = 27), lymph nodes (*n* = 23), sinuses (*n* = 21) and orbital cavity (*n* = 16) (Fig. 3).

Among these 108 reported patients with osseus RDD, a therapeutic approach was pursued in 69 individuals (Table 2). Within this group of patients, therapies included surgical interventions, carried out in 53 (76.8%) individuals, while glucocorticoid therapy was given to 20 (29.0%) patients. Less common interventions included other immunosuppressive drugs (*n* = 4, 5.8%, including methotrexate, azathioprine, cyclosporine A, mycophenolate mofetil, cyclophosphamide, infliximab and rituximab), chemotherapy (*n* = 8, 11.6%, including vinblastine, 6-mercaptopurine, vincristine, cladribine, clofarabine, chlorambucil, etoposide) or radiotherapy (*n* = 7, 10.1%). In a number of individuals, these therapeutic approaches were combined. In patients receiving initial therapy of any kind, total or partial remission was achieved in 54 (78.3%) individuals. In contrast, in a group of 13 (15.9%) patients with osseus RDD that did not receive any form of therapy, only four (30.8%) individuals achieved spontaneous remission. In 30 out of 108 cases, it was not mentioned whether patients received therapy or not. Twenty patients received a second-line therapy, six patients a third line, four patients a fourth line and only two a fifth-line therapy.

**Table 2:** Therapeutic approaches in osseous RDD

Patients with osseous RDD (*n* = 108)	Number (%)
Not documented	26
No therapy	13
Received therapy	69
Surgery	53 (76.8)
Glucocorticoids Chemotherapy Radiotherapy Immunosuppressive drugs Combination therapy	20 (29.0) 8 (11.6) 7 (10.1) 4 (5.8) 20 (29.0)

In osseous RDD, the involvement of lymph nodes had no significant impact whether or not a specific therapy was started. However, treatment was rather initiated depending on specific locations of RDD. Furthermore, additional nodal manifestation of RDD led to a more systemic treatment approach, whereas extranodal disease was treated in more than 50% by local surgical excision (Table 2).

## Summary

RDD is an extremely rare histiocytic disorder. Usually, patients present with painless swollen cervical lymph nodes. Additionally, they may have extranodal organ manifestation and isolated extranodal disease occurs as well. The clinical picture is heterogeneous and mainly determined by the localization of the disease. In particular, cranial manifestations and lesions affecting nervous structures can be problematic. Likewise, the clinical course is highly variable but there are several case reports suggesting spontaneous remissions of disease. Whether this means complete regression of all lesions or only clinical remission is unclear because patients rarely undergo follow-up biopsies.

Bone involvement is a rare manifestation of RDD occurring in roughly 10% of cases. Symptoms typically include pain and swelling. According to our systematic literature review, most affected patients are symptomatic and have one or two bone regions involved. There might be, however, a bias towards recognition of only symptomatic sites. Apparently, the cranial and facial bones as well as the long bones are the most affected sites. Involvement of the finger joints such as that in our patient seems to be rare. Up to three quarters of patients also have extraosseous involvement with predilection sites in the soft tissue, lymph nodes and sinuses. Thus, the head and neck regions seem to be primarily affected in RDD patients with bone involvement. Bone lesions in RDD are primarily osteolytic on conventional radiographs with some lesions also having sclerotic margins. Purely sclerotic lesions seem to be exceptionally rare.

In osseous RDD, most patients suffer from a chronic course of the disease. As detailed in the case reports, most patients therefore received some form of therapy. Primarily, patients underwent surgery or received glucocorticoids. A smaller proportion of patients underwent up to five different treatment regimens. Mostly, intensive treatment was related to disease manifestations of problematic organs such as those in the central nervous system, the vessels, the sinuses, the orbit and the nasal cavity. Spontaneous remissions were rarely reported. The clinical presentation of our patient fits well with what is known from the literature. She presented with mixed lytic and sclerotic bone lesions and suffered from a chronic disease course without spontaneous remission. Ongoing inflammation raised concerns about development of amyloidosis. Therefore, our patient received a combined therapy of surgical curettage of one lesion and immunomodulatory drug treatment with glucocorticoids, methotrexate and then rituximabas; single case reports described the positive effects of these immunomodulatory drugs in RDD [16, 17]. Over time, laboratory signs of inflammation weaned in our patient while lesions however persisted both in the lymph nodes and bone.

## Conclusion

Extranodal involvement in RDD is common but bone involvement occurs in less than 10% of cases. The true nature of RDD is yet unclear and there is no definitive clue for a clonal origin. Somatic mutations for instance in the BRAF gene (BRAF V600E mutation) have been described in ECD but not in RDD. Current evidence suggests a possible role for SLC29A3 mutations in familial RDD [18]. Overall, the optimal treatment of osseous RDD remains to be defined. Surgical resection in limited disease seems a valid option, while immunosuppressive treatments should be reserved for life- or organ-threatening cases. It is difficult to judge whether our immunomodulatory treatment approach had a significant effect on the course of the disease.

## Electronic supplementary material

ESM 1 (DOC 28 kb)

ESM 2 (DOCX 23 kb)

## References

[1] Foucar E, Rosai J, Dorfman R (1990). Sinus histiocytosis with massive lymphadenopathy (Rosai-Dorfman disease): review of the entity. Semin Diagn Pathol.

[2] McClain KL, Natkunam Y, Swerdlow SH. Atypical cellular disorders. Hematology Am Soc Hematol Educ Program. 2004:283–96. 10.1182/asheducation-2004.1.283 15561688

[3] Hervier B, Haroche J, Arnaud L, Charlotte F, Donadieu J, Neel A (2014). Association of both Langerhans cell histiocytosis and Erdheim-Chester disease linked to the BRAFV600E mutation. Blood.

[4] Weitzman S, Jaffe R (2005). Uncommon histiocytic disorders: the non-Langerhans cell histiocytoses. Pediatr Blood Cancer.

[5] • Emile JF, Abla O, Fraitag S, Horne A, Haroche J, Donadieu J, et al. Revised classification of histiocytoses and neoplasms of the macrophage-dendritic cell lineages. Blood. 2016. **Classification of RDD within histiocytic diseases, also giving insight into genetics.** 10.1182/blood-2016-01-690636 PMC5161007 26966089

[6] O’Malley DP, Duong A, Barry TS, Chen S, Hibbard MK, Ferry JA (2010). Co-occurrence of Langerhans cell histiocytosis and Rosai-Dorfman disease: possible relationship of two histiocytic disorders in rare cases. Mod Pathol.

[7] Dalia S, Sagatys E, Sokol L, Kubal T (2014). Rosai-Dorfman disease: tumor biology, clinical features, pathology, and treatment. Cancer Control.

[8] Rosai J, Dorfman RF (1969). Sinus histiocytosis with massive lymphadenopathy. A newly recognized benign clinicopathological entity. Arch Pathol.

[9] Kenn W, Eck M, Allolio B, Jakob F, Illg A, Marx A (2000). Erdheim-Chester disease: evidence for a disease entity different from Langerhans cell histiocytosis? Three cases with detailed radiological and immunohistochemical analysis. Hum Pathol.

[10] Menon MP, Evbuomwan MO, Rosai J, Jaffe ES, Pittaluga S (2014). A subset of Rosai-Dorfman disease cases show increased IgG4-positive plasma cells: another red herring or a true association with IgG4-related disease?. Histopathology.

[11] Rodriguez-Galindo C, Helton KJ, Sanchez ND, Rieman M, Jeng M, Wang W (2004). Extranodal Rosai-Dorfman disease in children. J Pediatr Hematol Oncol.

[12] Demicco EG, Rosenberg AE, Bjornsson J, Rybak LD, Unni KK, Nielsen GP (2010). Primary Rosai-Dorfman disease of bone: a clinicopathologic study of 15 cases. Am J Surg Pathol.

[13] Sundaram M, deMello D, Falbo S, Fallon RJ. Sinus histiocytosis with massive lymphadenopathy (Rosai-Dorfman disease) presenting with skeletal lesions. Skelet Radiol 1998;27(2):115–117. 10.1007/s002560050349 9526780

[14] Patterson FR, Rooney MT, Damron TA, Vermont AI, Hutchison RE (1997). Sclerotic lesion of the tibia without involvement of lymph nodes. Report of an unusual case of Rosai-Dorfman disease. J Bone Joint Surg Am.

[15] Bubolz AM, Weissinger SE, Stenzinger A, Arndt A, Steinestel K, Bruderlein S (2014). Potential clinical implications of BRAF mutations in histiocytic proliferations. Oncotarget.

[16] Pagel JM, Lionberger J, Gopal AK, Sabath DE, Loeb K (2007). Therapeutic use of rituximab for sinus histiocytosis with massive lymphadenopathy (Rosai-Dorfman disease). Am J Hematol.

[17] Inoue S, Onwuzurike N (2005). Venorelbine and methotrexate for the treatment of Rosai-Dorfman disease. Pediatr Blood Cancer.

[18] Morgan NV, Morris MR, Cangul H, Gleeson D, Straatman-Iwanowska A, Davies N (2010). Mutations in SLC29A3, encoding an equilibrative nucleoside transporter ENT3, cause a familial histiocytosis syndrome (Faisalabad histiocytosis) and familial Rosai-Dorfman disease. PLoS Genet.

