# Supplementary material for: Bone Involvement in Rosai-Dorfman Disease (RDD): a Case Report and Systematic Literature Review
Source: Curr Rheumatol Rep. 2017 Apr 11;19(5):29. doi: 10.1007/s11926-017-0656-6 (PMC5388731; doi:10.1007/s11926-017-0656-6)
Supplement: Supplementary file 1 — (DOC 28 kb) [file 11926_2017_656_MOESM1_ESM.doc]

Records excluded:

not-English literature or

bone involvement could be ruled out by the abstract

(n = 128)

Full-text articles excluded with reason:

no bone involvement (n=573) insufficient description of bone involvement (n=13)

Records identified through PubMed searching for “Rosai-Dorfman”, “Rosai and Dorfman” and “sinus histiocytosis with massive lymphadenopathy”

(n = 2210 )

Records after duplicated removed

(n = 802)

Full-text articles assessed for eligibility

(n =674)

Studies included in quantitative synthesis

(n =88)
