# Supplementary material for: Bone Involvement in Rosai-Dorfman Disease (RDD): a Case Report and Systematic Literature Review
Source: Curr Rheumatol Rep. 2017 Apr 11;19(5):29. doi: 10.1007/s11926-017-0656-6 (PMC5388731; doi:10.1007/s11926-017-0656-6)
Supplement: Supplementary file 2 — (DOCX 23 kb) [file 11926_2017_656_MOESM2_ESM.docx]

**Supplemetal References:**

1. Abdelwahab IF, Klein MJ, Springfield DS, Hermann G. A solitary lesion of talus with mixed sclerotic and lytic changes: Rosai-Dorfman disease of 25 years' duration. Skeletal Radiol. 2004;33(4):230-3.

2. Alawi F, Robinson BT, Carrasco L. Rosai-Dorfman disease of the mandible. Oral Surg Oral Med Oral Pathol Oral Radiol Endod. 2006;102(4):506-12.

3. Al-Saad K, Thorner P, Ngan BY, Gerstle JT, Kulkarni AV, Babyn P, et al. Extranodal Rosai-Dorfman disease with multifocal bone and epidural involvement causing recurrent spinal cord compression. Pediatr Dev Pathol. 2005;8(5):593-8.

4. Ambati S, Chamyan G, Restrepo R, Escalon E, Fort J, Pefkarou A, et al. Rosai-Dorfman disease following bone marrow transplantation for pre-B cell acute lymphoblastic leukemia. Pediatr Blood Cancer. 2008;51(3):433-5.

5. Bachmann KR, Dragoescu EA, Foster WC. Extranodal rosai-dorfman disease presenting as incidental bone tumor: a case report. Am J Orthop (Belle Mead NJ). 2010;39(11):E123-5.

6. Candeias da Silva C, Pedroso JL, Moraes FM, Rivero RL, Callegari FM, Araujo F, Jr., et al. Teaching NeuroImages: Rosai-Dorfman disease presenting with progressive early-onset cerebellar ataxia. Neurology. 2013;81(5):e27-8.

7. Chen HH, Zhou SH, Wang SQ, Teng XD, Fan J. Factors associated with recurrence and therapeutic strategies for sinonasal Rosai-Dorfman disease. Head Neck. 2012;34(10):1504-13.

8. Chen MW, King NK, Selvarajan S, Low DC. Benign scalp lump as an unusual presentation of extranodal Rosai-Dorfman disease. Surg Neurol Int. 2014;5:99.

9. Chen TH, Yang SN, Tseng HI. A lytic rib lesion in a neonate. J Paediatr Child Health. 2010;46(5):280-1.

10. Cossor F, Al-Khater AH, Doll DC. Laryngeal obstruction and hoarseness associated with Rosai-Dorfman disease. J Clin Oncol. 2006;24(12):1953-5.

11. Dean EM, Wittig JC, Vilalobos C, Garcia RA. A 16-year-old boy with multifocal, painless osseous lesions. Clin Orthop Relat Res. 2012;470(9):2640-5.

12. Demicco EG, Rosenberg AE, Bjornsson J, Rybak LD, Unni KK, Nielsen GP. Primary Rosai-Dorfman disease of bone: a clinicopathologic study of 15 cases. Am J Surg Pathol. 2010;34(9):1324-33.

13. Di Rocco F, Garnett MR, Puget S, Pueyerredon F, Roujeau T, Jaubert F, et al. Cerebral localization of Rosai-Dorfman disease in a child. Case report. J Neurosurg. 2007;107(2 Suppl):147-51.

14. Dickson-Gonzalez SM, Jimenez L, Barbella RA, Mota-Gamboa JD, Rodriguez-Morales AJ, Vals J, et al. Maxillofacial Rosai-Dorfman disease in a newly diagnosed HIV-infected patient. Int J Infect Dis. 2008;12(2):219-21.

15. Douleh DG, Carlson ML, Rinker EB, Haynes DS. Rosai-Dorfman Disease Involving the Ear and Lateral Skull Base. Otol Neurotol. 2015.

16. Duijsens HM, Vanhoenacker FM, ter Braak BP, Hogendoorn PC, Kroon HM. Primary intraosseous manifestation of Rosai-Dorfman disease: 2 cases and review of literature. JBR-BTR. 2014;97(2):84-9.

17. Geara AR, Ayoubi MA, Achram MC, Chamseddine NM. Rosai-Dorfman disease mimicking neurofibromatosis: case presentation and review of the literature. Clin Radiol. 2004;59(7):625-30.

18. George J, Stacy G, Peabody T, Montag A. Rosai-Dorfman disease manifesting as a solitary lesion of the radius in a 41-year-old woman. Skeletal Radiol. 2003;32(4):236-9.

19. Ghosal N, Furtado SV, Thakar S, Kumaran SP. Synchronous subcutaneous and calvarial Rosai-Dorfman disease diagnosed on fine needle aspiration cytology. Cytopathology. 2014;25(1):65-7.

20. Goel MM, Agarwal PK, Agarwal S. Primary Rosai-Dorfman disease of bone without lymphadenopathy diagnosed by fine needle aspiration cytology. A case report. Acta Cytol. 2003;47(6):1119-22.

21. Gupta K, Bagdi N, Sunitha P, Ghosal N. Isolated intracranial Rosai-Dorfman disease mimicking meningioma in a child: a case report and review of the literature. Br J Radiol. 2011;84(1003):e138-41.

22. Gupta P, Babyn P. Sinus histiocytosis with massive lymphadenopathy (Rosai-Dorfman disease): a clinicoradiological profile of three cases including two with skeletal disease. Pediatr Radiol. 2008;38(7):721-8; quiz 821-2.

23. Gupta S, Finzel KC, Grubber BL. Rosai-Dorfman disease masquerading as chronic ankle arthritis: a case report and review of the literature. Rheumatology (Oxford). 2004;43(6):811-2.

24. Hagemann M, Zbaren P, Stauffer E, Caversaccio M. Nasal and paranasal sinus manifestation of Rosai-Dorfman disease. Rhinology. 2005;43(3):229-32.

25. Hashimoto K, Kariya S, Onoda T, Ooue T, Yamashita Y, Naka K, et al. Rosai-Dorfman disease with extranodal involvement. Laryngoscope. 2014;124(3):701-4.

26. Hinduja A, Aguilar LG, Steineke T, Nochlin D, Landolfi JC. Rosai-Dorfman disease manifesting as intracranial and intraorbital lesion. J Neurooncol. 2009;92(1):117-20.

27. Hollon T, Camelo-Piragua SI, McKean EL, Sullivan SE, Garton HJ. Surgical Management of Skull Base Rosai-Dorfman Disease. World Neurosurg. 2016;87:661 e5- e12.

28. Hsu AR, Bhatia S, Kang RW, Arvanitis L, Nicholson GP, Virkus WW. Extranodal Rosai-Dorfman disease presenting as an isolated glenoid lesion in a high school athlete. J Shoulder Elbow Surg. 2012;21(1):e6-11.

29. Jing X, McHugh JB, Pu RT. Fine-needle aspiration cytology of Rosai-Dorfman disease of bone. Diagn Cytopathol. 2008;36(7):516-8.

30. Kademani D, Patel SG, Prasad ML, Huvos AG, Shah JP. Intraoral presentation of Rosai-Dorfman disease: a case report and review of the literature. Oral Surg Oral Med Oral Pathol Oral Radiol Endod. 2002;93(6):699-704.

31. Kang RW, McGill KC, Lin J, Gitelis S. Chronic ankle pain and swelling in a 25-year-old woman: an unusual case. Clin Orthop Relat Res. 2011;469(5):1517-21.

32. Kasapoglu Gunal E, Kamali S, Akdogan MF, Cimen AO, Ocal L, Agan M, et al. Rosai-Dorfman disease with factor XII deficiency. Clin Rheumatol. 2009;28(6):733-6.

33. Keskin A, Genc F, Gunhan O. Rosai-Dorfman disease involving maxilla: a case report. J Oral Maxillofac Surg. 2007;65(12):2563-8.

34. Khan AA, Siraj F, Rai D, Aggarwal S. Rosai-Dorfman disease of the paranasal sinuses and orbit. Hematol Oncol Stem Cell Ther. 2011;4(2):94-6.

35. Kidd DP, Revesz T, Miller NR. Rosai-Dorfman disease presenting with widespread intracranial and spinal cord involvement. Neurology. 2006;67(9):1551-5.

36. Kim do Y, Park JH, Shin DA, Yi S, Ha Y, Yoon do H, et al. Rosai-dorfman disease in thoracic spine: a rare case of compression fracture. Korean J Spine. 2014;11(3):198-201.

37. La Barge DV, 3rd, Salzman KL, Harnsberger HR, Ginsberg LE, Hamilton BE, Wiggins RH, 3rd, et al. Sinus histiocytosis with massive lymphadenopathy (Rosai-Dorfman disease): imaging manifestations in the head and neck. AJR Am J Roentgenol. 2008;191(6):W299-306.

38. Lauwers GY, Perez-Atayde A, Dorfman RF, Rosai J. The digestive system manifestations of Rosai-Dorfman disease (sinus histiocytosis with massive lymphadenopathy): review of 11 cases. Hum Pathol. 2000;31(3):380-5.

39. Le Guenno G, Galicier L, Fieschi C, Meignin V, Chabrol A, Oksenhendler E. Dramatic efficiency of pegylated interferon in sinus histiocytosis with massive lymphadenopathy. Br J Dermatol. 2011;164(1):213-5.

40. Li S, Yan Z, Jhala N, Jhala D. Fine needle aspiration diagnosis of Rosai-Dorfman disease in an osteolytic lesion of bone. Cytojournal. 2010;7:12.

41. Loh SY, Tan KB, Wong YS, Lee YS. Rosai-Dorfman disease of the triquetrum without lymphadenopathy. A case report. J Bone Joint Surg Am. 2004;86-A(3):595-8.

42. Lu CH, Chang KC, Lee EJ, Chuang MT, Chang RS. Intracranial Rosai-Dorfman disease with unusual transcranial extension. J Neuroimaging. 2012;22(3):312-5.

43. Lu M, Guo DY. Leptomeningeal Rosai-Dorfman disease. J Neuroradiol. 2010;37(3):196-7.

44. Ma J, Xiao J, Wang L. Extranodal Rosai-Dorfman disease with multilevel lumbar spinal lesions. J Neurosurg Spine. 2008;9(1):55-7.

45. Mannelli L, Monti S, Love JE, Kussick SJ, McLuen A, Behnia F. Primary Rosai-Dorfman disease of the bone in a patient with history of breast cancer: appearance on 99mTc-MDP scintigraphy, CT, and X-ray. Clin Nucl Med. 2015;40(3):247-9.

46. Maratos EC, Bridges LR, MacKinnon AD, Madigan JB, Atra A, Martin AJ. Isolated intracranial Rosai-Dorfman disease in a child, a case report and review of the literature. Childs Nerv Syst. 2014;30(9):1595-600.

47. Miyake M, Tateishi U, Maeda T, Arai Y, Sugimura K, Hasegawa T. Extranodal Rosai-Dorfman disease: a solitary lesion with soft tissue reaction. Radiat Med. 2005;23(6):439-42.

48. Mohadjer Y, Holds JB, Rootman J, Wilson MW, Gigantelli JW, Custer PL. The spectrum of orbital Rosai-Dorfman disease. Ophthal Plast Reconstr Surg. 2006;22(3):163-8.

49. Mota Gamboa JD, Caleiras E, Rosas-Uribe A. Extranodal Rosai-Dorfman disease. Clinical and pathological characteristics in a patient with a pseudotumor of bone. Pathol Res Pract. 2004;200(5):423-6; discussion 7-8.

50. Ojha J, McIlwain R, Said-Al Naief N. A large radiolucent lesion of the posterior maxilla. Oral Surg Oral Med Oral Pathol Oral Radiol Endod. 2010;110(4):423-9.

51. Oner AY, Akpek S, Tali T. Rosai-Dorfman disease with epidural and spinal bone marrow involvement: magnetic resonance imaging and diffusion-weighted imaging features. Acta Radiol. 2007;48(3):331-4.

52. Ortonne N, Fillet AM, Kosuge H, Bagot M, Frances C, Wechsler J. Cutaneous Destombes-Rosai-Dorfman disease: absence of detection of HHV-6 and HHV-8 in skin. J Cutan Pathol. 2002;29(2):113-8.

53. Orvets ND, Mayerson JL, Wakely PE, Jr. Extranodal Rosai-Dorfman disease as solitary lesion of the tibia in a 56-year-old woman. Am J Orthop (Belle Mead NJ). 2013;42(9):420-2.

54. P OR, Patel V, Luthert P, Chandrasekharan L, Malhotra R. Orbital Rosai-Dorfman disease with subperiosteal bone involvement mimicking eosinophilic granuloma. Orbit. 2012;31(1):24-6.

55. Parida PK, Panda NK, Sharma A, Mahesha V, Das A. Varied manifestations of Rosai-Dorfman disease and its management - a case report and review of literature. Indian J Otolaryngol Head Neck Surg. 2008;60(4):365-8.

56. Patel JN, Wang WL, Murphy WA, Jr. Painful left shoulder. Extranodal primary osseous form of Rosai-Dorfman disease. Skeletal Radiol. 2012;41(11):1463-4, 89-90.

57. Patel MH, Jambhekar KR, Pandey T, Ram R. A rare case of extra nodal Rosai-Dorfman disease with isolated multifocal osseous manifestation. Indian J Radiol Imaging. 2015;25(3):284-7.

58. Petzold A, Thom M, Powell M, Plant GT. Relapsing intracranial Rosai-Dorfman disease. J Neurol Neurosurg Psychiatry. 2001;71(4):538-41.

59. Prabhakaran VC, Bhatnagar A, Sandilla J, Olver J, Leibovitch I, Ghabrial R, et al. Orbital and adnexal Rosai-Dorfman disease. Orbit. 2008;27(5):356-62.

60. Purav P, Ganapathy K, Mallikarjuna VS, Annapurneswari S, Kalyanaraman S, Reginald J, et al. Rosai-Dorfman disease of the central nervous system. J Clin Neurosci. 2005;12(6):656-9.

61. Ramadass T, Das Thulasi P, Geetha N, Narayanan N, Ayyaswamy G, Swapna S. Extranodal manifestation of Rosai Dorfman Disease of the nasopharynx. Indian J Otolaryngol Head Neck Surg. 2007;59(2):178-81.

62. Rittner RE, Baumann U, Laenger F, Hartung D, Rosenthal H, Hueper K. Whole-body diffusion-weighted MRI in a case of Rosai-Dorfman disease with exclusive multifocal skeletal involvement. Skeletal Radiol. 2012;41(6):709-13.

63. Robert EG, Fallon KB, Tender GC. Isolated Rosai-Dorfman disease of the sacrum. Case illustration. J Neurosurg Spine. 2006;4(5):425.

64. Rodriguez-Galindo C, Helton KJ, Sanchez ND, Rieman M, Jeng M, Wang W. Extranodal Rosai-Dorfman disease in children. J Pediatr Hematol Oncol. 2004;26(1):19-24.

65. Safdar A, Gillenwater AM, Jones DM, Jorgensen JL, Romaguera JE. Rosai-Dorfman disease misdiagnosed as active tuberculosis. Leuk Lymphoma. 2006;47(7):1441-2.

66. Sasaki K, Pemmaraju N, Westin JR, Wang WL, Khoury JD, Podoloff DA, et al. A single case of rosai-dorfman disease marked by pathologic fractures, kidney failure, and liver cirrhosis treated with single-agent cladribine. Front Oncol. 2014;4:297.

67. Schein C, Kluskens L, Gattuso P. Fine-needle aspiration of primary Rosai-Dorfman disease of the bone without peripheral lymphadenopathy: a challenging diagnosis. Diagn Cytopathol. 2013;41(3):230-1.

68. Sciacca S, Barkas K, Heptinstall L, McNamara C, Shetty R. Rosai-Dorfman disease with spinal cord compression: a diagnostic challenge. Eur Spine J. 2015;24 Suppl 4:S529-35.

69. Scumpia AJ, Frederic JA, Cohen AJ, Bania M, Hameed A, Xiao PQ. Isolated intracranial Rosai-Dorfman disease with orbital extension. J Clin Neurosci. 2009;16(8):1108-9.

70. Sellari-Franceschini S, Lenzi R, Tognetti A, Seccia V. Extranodal Rosai-Dorfman disease of bone and nose: a case report and review of literature. Pathologica. 2010;102(2):62-6.

71. Setareh M, Zahra M, Vahid M, Farah S. Generalized lymphadenopathy in infancy; a case report. Iran J Pediatr. 2013;23(1):105-8.

72. Shi Y, Griffin AC, Zhang PJ, Palmer JN, Gupta P. Sinus histiocytosis with massive lymphadenopathy (Rosai-Dorfman Disease): A case report and review of 49 cases with fine needle aspiration cytology. Cytojournal. 2011;8:3.

73. Shulman S, Katzenstein H, Abramowsky C, Broecker J, Wulkan M, Shehata B. Unusual presentation of Rosai-Dorfman disease (RDD) in the bone in adolescents. Fetal Pediatr Pathol. 2011;30(6):442-7.

74. Simko SJ, Tran HD, Jones J, Bilgi M, Beaupin LK, Coulter D, et al. Clofarabine salvage therapy in refractory multifocal histiocytic disorders, including Langerhans cell histiocytosis, juvenile xanthogranuloma and Rosai-Dorfman disease. Pediatr Blood Cancer. 2014;61(3):479-87.

75. Sundaram C, Uppin Shantveer G, Chandrashekar P, Prasad VB, Umadevi M. Multifocal osseous involvement as the sole manifestation of Rosai-Dorfman disease. Skeletal Radiol. 2005;34(10):658-64.

76. Tasso M, Esquembre C, Blanco E, Moscardo C, Niveiro M, Paya A. Sinus histiocytosis with massive lymphadenopathy (Rosai-Dorfman disease) treated with 2-chlorodeoxyadenosine. Pediatr Blood Cancer. 2006;47(5):612-5.

77. Tekin U, Tuz HH, Gunhan O. Reconstruction of a patient with Rosai-Dorfman disease using ramus graft and osseointegrated implants: a case report. J Oral Implantol. 2012;38(1):79-83.

78. Thavaraj V, Dawar R, Arya LS. Sinus histiocytosis with massive lymphadenopathy in children. Indian Pediatr. 2002;39(8):764-9.

79. Tsang JS, Anthony MP, Wong MP, Wong CS. The use of FDG-PET/CT in extranodal Rosai-Dorfman disease of bone. Skeletal Radiol. 2012;41(6):715-7.

80. Tubbs RS, Kelly DR, Mroczek-Musulman EC, Hammers YA, Berkow RL, Oakes WJ, et al. Spinal cord compression as a result of Rosai-Dorfman disease of the upper cervical spine in a child. Childs Nerv Syst. 2005;21(11):951-4.

81. Ture U, Seker A, Bozkurt SU, Uneri C, Sav A, Pamir MN. Giant intracranial Rosai-Dorfman disease. J Clin Neurosci. 2004;11(5):563-6.

82. Walczak BE, Halperin DM, Bdeir RW, Irwin RB. Orthopaedic case of the month: a 50-year-old woman with persistent knee pain. Clin Orthop Relat Res. 2011;469(12):3527-32.

83. Walid MS, Grigorian AA. Ethmo-spheno-intracranial Rosai-Dorfman disease. Indian J Cancer. 2010;47(1):80-1.

84. Wrzolek MA, Zagzag D. May 2002: 38-year-old man and 69-year-old woman with dural based masses. Brain Pathol. 2002;12(4):517-8, 21.

85. Wu F, Hao SP. Rosai-Dorfman disease presented as saddle nose. Otolaryngol Head Neck Surg. 2008;138(1):124-5.

86. Wu SY, Ma L, Tsai YJ. Partial removal of orbital tumor in Rosai-Dorfman disease. Jpn J Ophthalmol. 2004;48(2):154-7.

87. Yoon AJ, Parisien M, Feldman F, Young-In Lee F. Extranodal Rosai-Dorfman disease of bone, subcutaneous tissue and paranasal sinus mucosa with a review of its pathogenesis. Skeletal Radiol. 2005;34(10):653-7.

88. Yuen HK, Cheuk W, Leung DY, Tse RK, Chan N. Atypical presentation of Rosai-Dorfman disease in the lacrimal gland mimicking malignancy. Ophthal Plast Reconstr Surg. 2006;22(2):145-7.
